# Evaluation of the systemic micro- and macrovasculature in stable angina: A case-control study

**DOI:** 10.1371/journal.pone.0178412

**Published:** 2017-05-25

**Authors:** Ulf Neisius, Erin Olson, Sabrina H. Rossi, Hagar A. Ibrahim, Gemma Currie, Anna F. Dominiczak, Christian Delles

**Affiliations:** 1 BHF Glasgow Cardiovascular Research Centre, Institute of Cardiovascular and Medical Sciences, University of Glasgow, Glasgow, United Kingdom; 2 Department of Medicine, Cardiovascular Division, Beth Israel Deaconess Medical Center, Harvard Medical School, Boston, Massachusetts, United States of America; Nagoya University, JAPAN

## Abstract

**Aims:**

The diagnosis of stable angina involves the use of probability estimates based on clinical presentation, age, gender and cardiovascular risk factors. In view of the link between the cardiac and systemic vasculature we tested whether non-invasive measures of systemic micro- and macrovascular structure and function differentiate between individuals with flow-limiting coronary artery disease (CAD) and those with normal coronary arteries (NCA).

**Methods and results:**

We recruited 84 patients undergoing elective coronary angiography for investigation of symptoms of stable angina. Patients were selected for either having significant CAD or NCA (n = 43/41; age, 56±7 *vs* 57±7 years, *P* = 0.309). Only microvascular endothelial function, measured using the Endo-PAT2000 device to determine reactive hyperaemia index (CAD vs. NCA; 1.9 [1.5; 2.3] vs. 2.1 [1.8; 2.4], P = 0.03) and sonographic carotid plaque score (CAD vs. NCA; 3.0 [1.5; 4.5] vs. 1.2 [0; 2.55], P<0.001) were significantly different between patients with CAD and NCA. No significant differences were detected in reflection magnitude (CAD vs. NCA; 1.7 [1.5; 1.8] % vs 1.7 [1.5; 1.9] %, *P* = 0.342), pulse wave velocity (CAD vs. NCA; 7.8±1.4 m/sec vs. 8.3±1.5 m/sec, P = 0.186), carotid intima-media thickness (CAD vs. NCA; 0.73±0.10 mm vs. 0.75±0.10 mm, *P* = 0.518) or carotid distensibility (CAD vs. NCA; 3.8±1.2 10-3/kPa vs. 3.4±0.9 10-3/kPa, P = 0.092). Also, the c-statistic of the pre-test probability based on history and traditional risk factors (c = 0.665; 95% CI, 0.540–0.789) was improved by the addition of the inverse RHI (c = 0.720; 95% CI, 0.605–0.836), carotid plaque score (c = 0.770, 95% CI, 0.659–0.881), and of both markers in combination (c = 0.801; 95% CI, 0.701–0.900).

**Conclusion:**

There are distinct differences in the systemic vasculature between patients with CAD and NCA that may have the potential to guide diagnostic and therapeutic decisions. Carotid artery plaque burden and microvascular function appear to be most promising in this context.

## Introduction

Stable angina is a common disorder with an estimated prevalence of 2–4% in the Western world [1]. In patients describing symptoms suggestive of stable angina probability estimation is used to triage further diagnostic procedures [1,2]. This pre-test probability is usually based on the type of chest pain, age and gender [3], but can be further adjusted with resting ECG findings and cardiovascular risk factors [4,5]. Refining coronary artery disease (CAD) estimation could reduce the number of false positive stress test results and consecutively minimise the number of potentially harmful invasive investigation by exclusion of patients with pre-test probability such as <5% [1] or <15% [2] from further testing. Differences in systemic vascular markers between patients with/without CAD are well established [6–14]. Several cohort studies that assessed carotid intima-media thickness (C-IMT) and plaque [7–11] or endothelial function [12–14] suggested a potential role for these tests in improving CAD risk stratification. Most studies, however, have not systematically assessed a range of functional and structural markers in small and large vessels simultaneously. A large number of tests and devices are available for the assessment of the systemic vasculature [6]. Their value in risk prediction and especially in CAD estimation is subject to debate. We chose to assess six markers in order to obtain a snapshot of functional and structural properties of the systemic circulation in the development of cardiovascular disease [15].

## Material and methods

The study was designed as a two-step approach (S1 Fig). The primary aim was to identify vascular markers differentiating between chest pain patients with flow-limiting CAD and normal coronary arteries (NCA) and emerging from these results the investigation of their potential contribution to probability estimation. The secondary aim was to assess their potential to refine the estimation of post- (stress) test probability.

The study recruitment has been described in detail elsewhere [16] and is summarized in S1 Fig. Local cardiologists referred patients (n = 2045) with angina symptoms and/or positive non-invasive tests to the Golden Jubilee National Hospital, Clydebank, UK for elective coronary angiography between January 2009 and August 2010. We selected retrospectively those with significant CAD, defined as a luminal obstruction of the left main stem ≥50% and/or at least one coronary artery stenosis ≥75% of the remaining coronary artery lumen; or NCA, defined as the absence of artery narrowing on coronary angiography for non-cardiac vascular assessment. Information regarding the results of coronary angiography was retrieved from a local database, where the degree of atherosclerosis was evaluated visually by the cardiologist performing the angiography. Study visits took place an average of 285 (58 to 459) days after coronary angiography.

Basic assessment during the study visit included clinical and demographic data. Information regarding cardiovascular risk factors was acquired from interviews, medical records or direct measurements. In this context, hypertension was defined as blood pressure ≥140/90 mmHg on two occasions, treatment with at least one antihypertensive drug, or history of hypertension according to medical records and/or the study visit interview. Hyperlipidaemia was defined as either fasting cholesterol of ≥5.5 mmol/l and/or a written or verbal history of hyperlipidaemia. Blood samples were analyzed for cholesterol levels at the study visit. Diabetes was defined in accordance with medical records or self-reporting during the study visit. Current smoking status was considered positive when study participants smoked during the week prior to the study visit. A positive family history was defined as prevalence of symptomatic CAD in at least one 1^st^ degree family member.

All participants gave written informed consent. The study was approved by the West of Scotland Research Ethics Committee and is in keeping with the principles of the Declaration of Helsinki.

**Fig 1:**
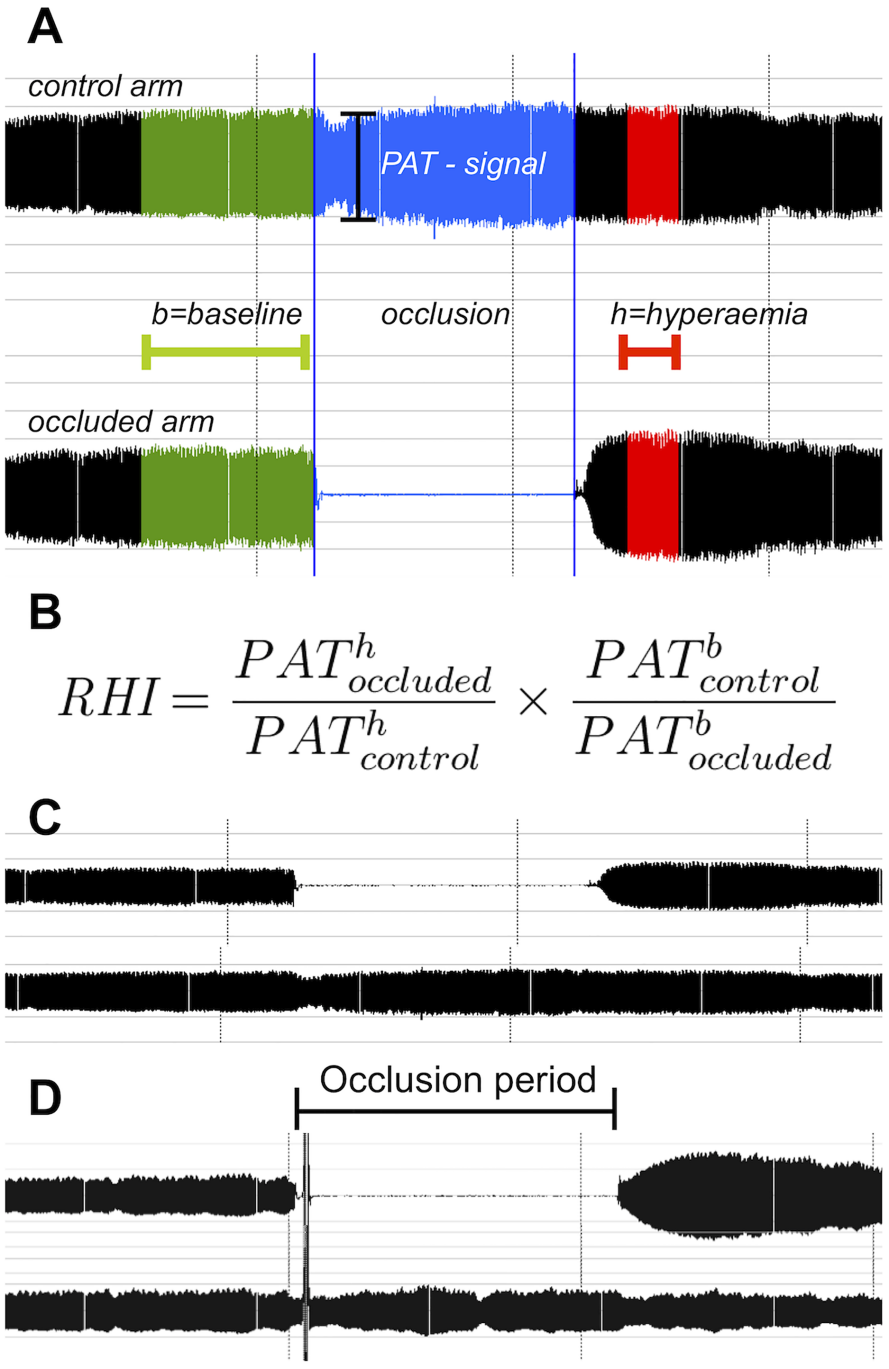
Illustration of reactive hyperaemia peripheral arterial tonometry (PAT). Depicted are a measurement (A) with colour-coded intervals corresponding to parameters required for the reactive hyperaemia index equation (B). Tracings derive from beat-to-beat finger volume changes captured by plethysmographic finger cuffs on the index fingers of both hands. The interval 60–120 seconds post cuff release is best linked to coronary microvasculature dysfunction as published by Bonnetti et al. [17]. Additionally the comparison of a pathologic (C) and a normal (D) hyperaemia responses as recorded by fingertip plethysmography is shown. RHI, reactive hyperaemia index.

Endothelial function was assessed non-invasive and non-pharmacological with peripheral arterial tonometry (PAT) performed with the Endo-PAT2000 device (Itamar Medical Ltd., Caesarea, Israel). Blood pressure and heart rate were measured 3 times with 2-minute intervals before the examination. The mean of the last 2 recordings was used as the baseline blood pressure and heart rate. Beat-to-beat finger volume changes were captured by single-use plethysmographic finger cuffs on the index fingers of both hands. After an equilibration period of at least 5 minutes to assess the baseline PAT signal a blood pressure cuff (Hokanson SC12, Bellevue, USA) was inflated to at least 200 mmHg or pressures 60 mmHg above the systolic blood pressure for exactly five minutes on the upper right arm. To ascertain that the brachial artery was completely occluded, the on screen scale of the PAT signal was increased to maximum amplitude. The cuff pressure was then released and the hyperaemia PAT signal was recorded, as shown in Fig 1. Measurement from the contra-lateral arm were used to control for changes in vascular tone. The digital pulse amplitude was continually obtained and digitally recorded to a laptop. The data was analyzed by a a computerized algorithm, which operator independent and automatically calculates the reactive hyperaemia index (RHI). The RHI represents the ratio between the PAT signal after hyperaemia and at baseline adjusted for the control finger. The corresponding equation is illustrated in Fig 1. To avoid interference of factors such as outside temperature, physical activity, drug intake and smoking, recordings were performed in a temperature controlled room at 23–24°C, after 20 minutes of rest in supine position. Participants were fasted and refrained from smoking for at least 2 hours.

Following we will briefly describe the five remaining non-invasive, non-pharmacological vascular measurements. All tests, including endothelial function assessment, were conducted during a single study visit within a time frame of approximately 120 minutes and under identical environmental conditions. The order of investigations was as follows: electrocardiogram (not reported), pulse wave velocity, pulse wave analysis, and endothelial function in one room followed or proceeded by carotid ultrasound in another room. Further detail is provided in the supplementary material (S1 File and S2–S4 Figs).

Radial arterial pressure waveforms were recorded with a Millar piezo-resistive pressure transducer (Millar SPT 301, Millar Instruments, Houston, US) coupled to a SphygmoCor device (AtCor Medical, Sydney, Australia). The corresponding central (ascending aortic) waveform was generated by the SphygmoCor software (version 7.0).

To quantify the maximum of the forward and backward pressure waves in the aortic root the triangulation method as reported by Westerhof et al. [18] was used based on central pressure curve produced by the SphygmoCor device. To adjust for pressure wave extent reflection magnitude was calculated as the ratio of the backward and forward pressure amplitudes.

Carotid femoral pulse wave velocity (PWV) was measured using the SphygmoCor device according to the manufacturer’s protocol. Further detail can be found in the S1 File.

Measurement of C-IMT was performed by ultrasonography (Acuson Sequoia C512, Siemens, Erlangen, Germany) with an 8 MHz linear-array transducer, in accordance with the Mannheim consensus [19]. Offline measurements were performed semi-automatically at end diastole on B-mode images using Image-Pro Plus software, version 3.0 (Media Cybernetics, Bethesda, USA).

Offline B-mode common carotid artery images were assessed for plaque presence and extent in correspondence with the Mannheim consensus [19] Plaque score was then calculated as described by Hollander et al. [20] and van der Meer et al. [21] (S2 Fig).

Diameter changes in the common carotid arteries during the cardiac cycle were recorded to assess carotid distensibility via M-mode imaging and were measured offline with Image-Pro Plus software, version 3.0 (Media Cybernetics, Bethesda, USA). The average of the left and right common carotid artery measurements was used for calculation of the distensibility coefficient (S3 Fig).

Results from exercise tolerance tests (n = 75) and myocardial perfusion single-photon emission computed tomography scans (n = 10) prior to coronary angiography were extracted from medical records. Exercise tolerance testing following the standard Bruce protocol was performed on locally available treadmills at different hospitals in Glasgow. A positive exercise test result was defined in accordance with clinical guidelines [22].

Data were analysed using SPSS software, version 21.0 (IBM Corp., Armonk, USA). Normality of data distribution was determined using the Kolmogorov-Smirnov test and visual inspection of Q-Q plots. The two-sample Student’s t test or the Mann Whitney U-test were conducted as appropriate for comparison of continuous variables between groups. For comparison of categorical data the Chi-squared test was employed.

The algorithm developed by Pryor et al. [4,5] was used to estimate pre-test probability of flow-limiting CAD (S1 File). As defined by the Bayesian theory, the post-test likelihood of flow-limiting CAD after a functional test is heavily influenced by the pre-test probability estimate. Therefore the post-test probabilities were calculated multiplying the pre-test probability with likelihood ratios [23]. The latter were computed with published test sensitivities and specificities [24].

Other reported pre-test probability estimates were derived from synoptical tables published by Diamond and Forrester [3], Fihn et al. [1], and Genders et al. [4–5], which reference angina typology, age and gender.

A stepwise binary logistic regression model for the prediction of CAD was used to calculate the positive predictive value of different micro- and macrovascular parameters in combination with pre- and post-test probabilities. The presence/absence of CAD was used as the outcome variable and ≥1 vascular markers plus pre- or post-test probabilities as independent variables. Receiver operating characteristic (ROC) curves were drawn using the resulting predictive values. Such ROC curves depict the performance of a binary classifier system while its discriminatory threshold is varied and where each point represents a sensitivity/specificity pair for a particular threshold value.

## Results

Based on the study inclusion criteria both subgroups were well matched for anthropometric features and cardiovascular risk profiles, as shown in Table 1. The gender mismatch relates to disease prevalence and contributed significantly to CAD estimation [4,5] and consecutively to post-test probability values. Evaluation of the pre-test probability according to symptoms, age and gender [10] showed that most patients were in the high or intermediate CAD estimate categories.

**Table 1:** Demographic data and angina history.

	CAD (n = 43)	NCA (n = 41)	*P*-Value
Gender, m/f	26/17	13/28	0.008
Age, y	55.7±7.0	57.2±7.1	0.309
Height, cm	167±8	167±12	0.923
Weight, kg	78±14	78±23	0.945
BMI, kg/m^2^	28.1±4.1	27.8±6.6	0.839
WHR	0.91±0.08	0.92±0.07	0.763
SBP, mmHg	136±19	137±18	0.779
DBP, mmHg	78±10	81±9	0.282
ACEI, %	44	29	0.155
CCB, %	28	26	0.912
BB, %	91	22	<0.001
Statin, %	91	49	<0.001
Fibrates, %	2	0	-
Diabetes, %	23	17	0.280
Hypertension, %	65	59	0.615
Hyperlipidaemia, %	46	54	0.497
Positive family history, %	74	68	0.534
Current Smoker, %	28	17	0.516
Stroke/TIA, %	0	0	-
Typical angina, %	70	59	0.529
Atypical angina, %	26	29	0.933
Nonanginal chest pain, %	4	12	0.434
Probability estimate categories (Diamond/Forrester) [3]			0.479
>90%, n	23	15	
10–90%, n	17	22	
<10%, n	2	3	
<5%, n	1	1	
Probability of flow limiting CAD (Pryor et al.) [4,5]	0.69 [0.55;0.89]	0.53 [0.27;0.67]	<0.001
Pre-test Likelihood of flow limiting CAD (Combined Diamond/Forrester and CASS data) [1], %	68.23±27.06	59.66±28.68	0.179
Pre-test Likelihood of flow limiting CAD (Genders et al.) [5,25], %	54.21±23.58	43.90±21.58	0.040

ACEI, Angiontensin converting enzyme inhibitor; BB, beta blocker; CAD, coronary artery disease; BMI, body mass index; CCB, calcium channel blocker; DBP, diastolic blood pressure; ESC, European Society of Cardiology; f, female; m, male; NCA, normal coronary arteries; SBP, systolic blood pressure; TIA, transitory ischaemic attack; vasoactive substance, summary of nicorandil, oral nitrate and CCB therapy; WHR, waist-to-hip ratio.

Differences in medication between the groups were due to the retrospective nature of the study, as well as therapeutic indication in patients with confirmed CAD [1,2]. Therefore patients with CAD were more frequently treated with statins and anti-anginal medication.

Exercise tolerance test results were available for 75 participants. According to current diagnostic criteria 46 participants had positive results (27:19, CAD vs. NCA) [22]. Myocardial perfusion single-photon emission computed tomography scans showed inducible perfusion defects in five additional patients with NCA. There was no significant difference in positive functional stress tests between the two groups (CAD:NCA, 27:12 vs. 24:14, P = 0.573). However the calculation of post-test probabilities revealed a significant difference (CAD:NCA; 1.55±1.05 vs. 0.94±0.79, P = 0.005).

**Fig 2:**
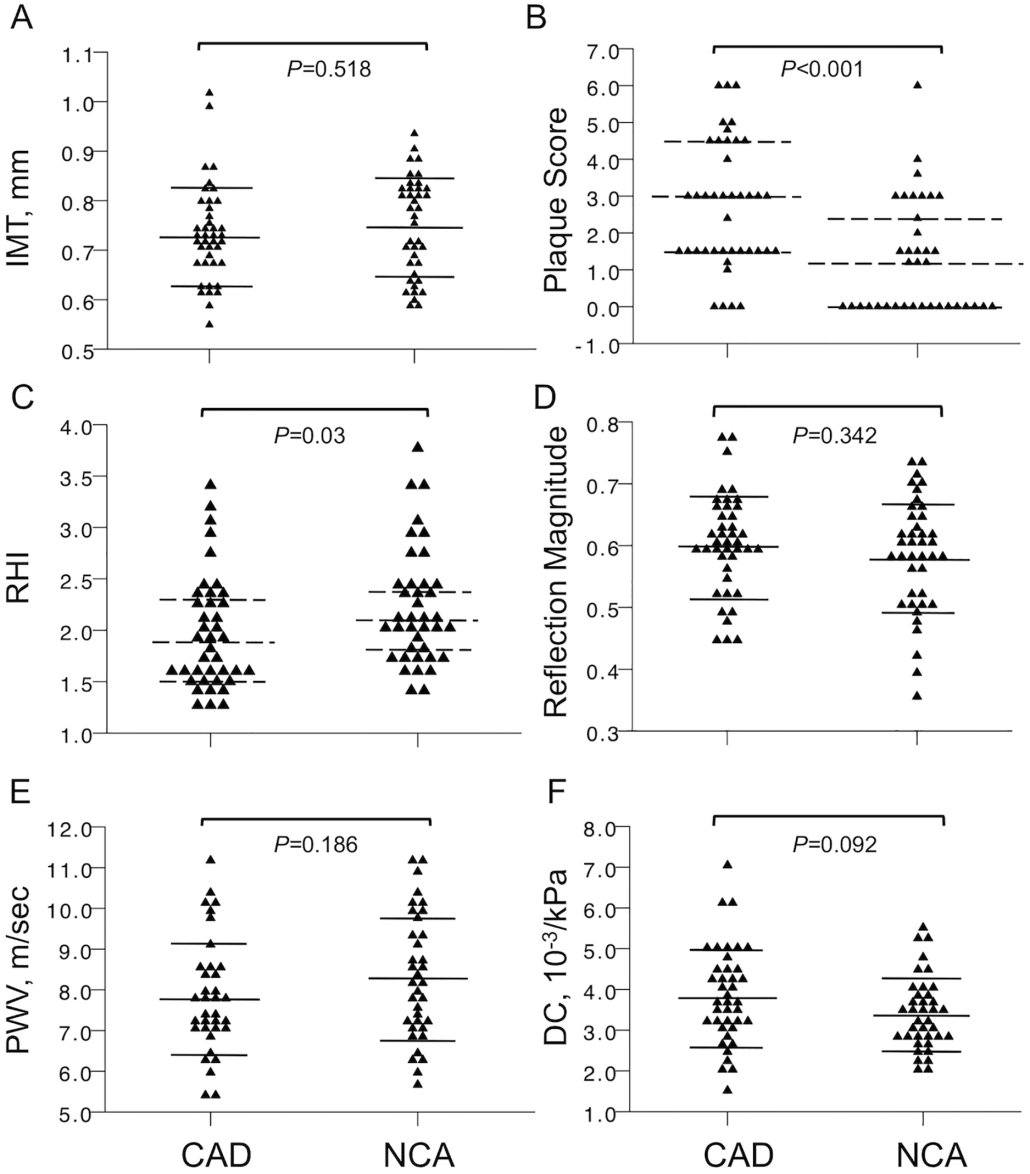
Non-cardiac vascular markers in patients with angina like symptoms and CAD or normal coronary arteries. Illustrated are carotid IMT (A), plaque score (B), reactive hyperaemia index (C), reflection magnitude (D), pulse wave velocity (E) and carotid distensibility (F). The continuous lines represent the mean±SD (————) and the dotted lines represent the median + interquartile range (— — —). CAD, coronary artery disease; NCA, normal coronary arteries; IMT, carotid intima media thickness; RHI, reactive hyperaemia index; PWV, pulse wave velocity; DC, distensibility coefficient; ns, non-significant.

Carotid plaque score (CAD:NCA, n = 41:37; 3.0 [1.5; 4.5] vs. 1.2 [0; 2.55], P<0.001) was the only significantly different macrovascular marker between the CAD and NCA groups (Fig 2B). Markers of arterial stiffness such as carotid distensibility (CAD vs. NCA; 3.8±1.2 10-3/kPa vs. 3.4±0.9 10-3/kPa, *P* = 0.092) and pulse wave velocity (CAD vs. NCA; 7.8±1.4 m/sec vs. 8.3±1.5 m/sec, *P* = 0.186); or of generalized atherosclerosis such as C-IMT (CAD vs. NCA; 0.73±0.10 mm vs. 0.75±0.10 mm, *P* = 0.518) were not significantly different (Fig 1). Other macrovascular markers are listed in S1 Table.

Microvascular assessment showed a significant difference in endothelial function where RHI values in NCA patients were indicative of a healthier endothelium (CAD vs. NCA; 1.9 [1.5; 2.3] vs. 2.1 [1.8; 2.4], *P* = 0.03) (Fig 2C). This finding persisted when only patients with positive stress tests were investigated (CAD vs. NCA; 1.9 [1.6; 2.3] vs. 2.1 [2.0; 2.5], *P* = 0.02). Resistance vessel function assessed by the reflection magnitude (CAD vs. NCA; 0.60±0.08 vs. 0.58±0.09, *P* = 0.342) was however not significantly different.

**Fig 3:**
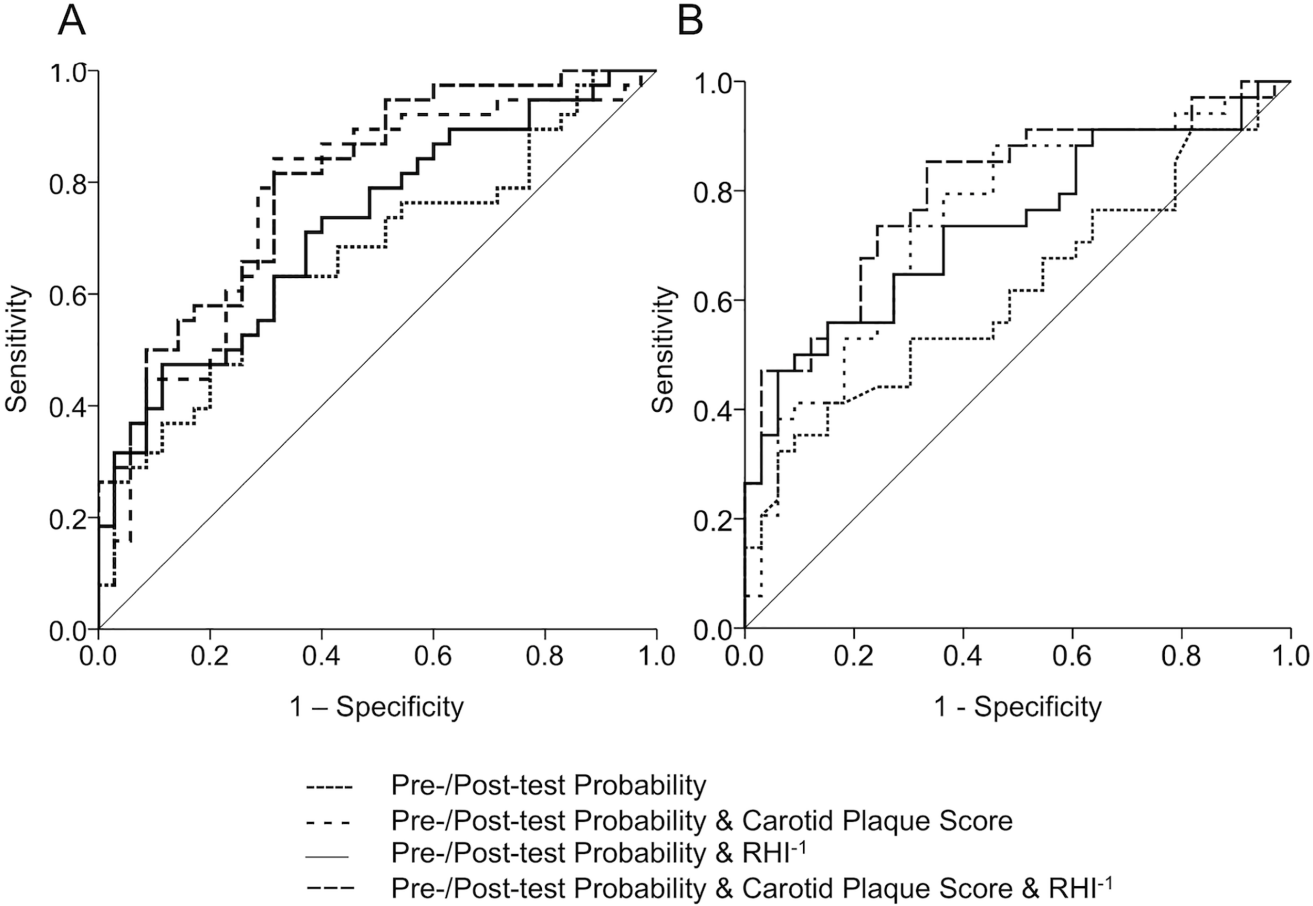
ROC-analysis for CAD estimation. Depicted are ROC-curves for pre- (A) and post-test probability (B) and added vascular markers. RHI^-1^, inverse reactive hyperaemia index.

The majority of participants underwent assessments of endothelial function, carotid plaque score, pre-test probability (n = 73) and post-test probability (n = 67). ROC analysis for estimation of pre-test probability (c = 0.665; 95% CI, 0.540–0.789) was improved by the addition of the inverse RHI (c = 0.720; 95% CI, 0.605–0.836), carotid plaque score (c = 0.770, 95% CI, 0.659–0.881), and of both markers in combination (c = 0.801; 95% CI, 0.701–0.900) (Fig 3A). Similarly, ROC analysis for estimation of post-test probability (c = 0.617; 95% CI, 0.487–0.753) was improved by the addition of the inverse RHI (c = 0.737; 95% CI, 0.617–0.857), of the carotid plaque score (c = 0.745; 95% CI, 0.626–0.864) and of both markers (c = 0.800; 95% CI 0.694–0.907) (Fig 3B). The inverse RHI was used to adjust for the negative correlation of index values with endothelial function. In logistic regression analyses the combination of RHI and carotid plaque score in addition to pre- (pre-test probability/plaque score/RHI; *χ*^2^ = 20.8571, df = 3, *P* = 0.0001; B = 1.6087/0.6056/-1.2366, *P* = 0.1904/0.0019/0.0280, SE = 1.2284/0.1951/0.5626, OR = 4.9962/1.8323/ 0.2904) or post-test probabitity (post-test probability/plaque score/RHI; *χ*2 = 19.6582, df = 3, *P* = 0.0002; B = 0.3685/0.5859/-1.6280, *P* = 0.2477/0.0033/ 0.0114, SE = 0.3188/ 0.1992/0.6436, OR = 1.4456/1.7966/0.1963) contributed significantly to the prediction of CAD.

Considering the plaque score distribution in Fig 2B, we calculated the test characteristics for carotid plaque vs. no carotid plaque in our cohort (CAD vs. NCA; plaque detected/no plaque, n = 37/4 vs. n = 19/18, *P*<0.001; Positive Predictive Value = 66%, Negative Predictive Value = 82%, Sensitivity = 90%, Specificity = 49%).

## Discussion

**Fig 4:**
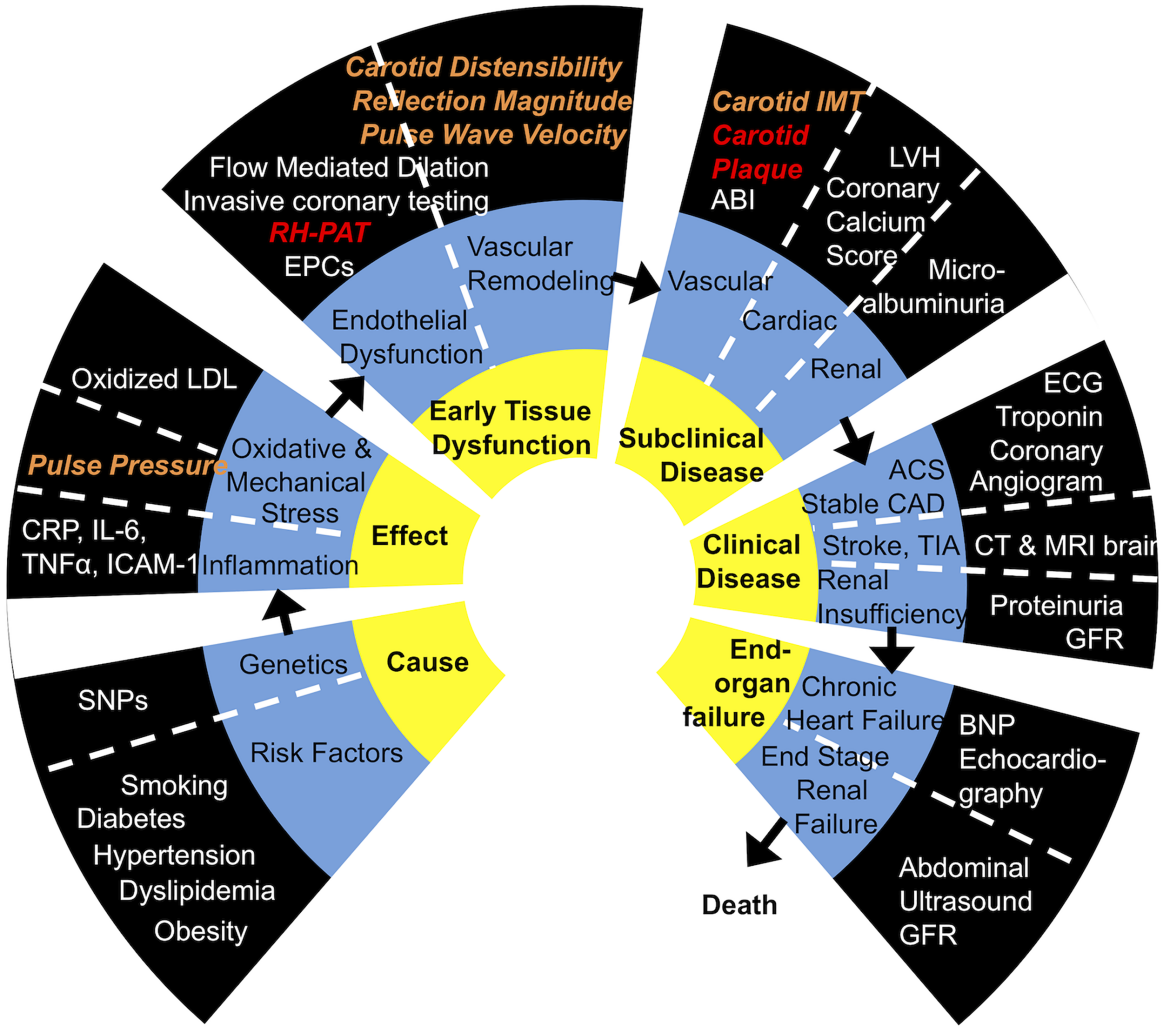
Markers in the cardiovascular continuum. The inner circle (yellow) states the stage of the cardiovascular continuum. The middle circle (blue) states the different pathophysiological process or categorises of the cardiovascular continuum stage. The outer circle (black) summarises a range of markers quantifying the pathophysiological process. Investigated markers (***bold & italic***) capable of differentiating between patients with CAD and NCA in our study are highlighted red. Investigated markers (***bold & italic***) without statistical significant differences between patients with CAD and NCA are highlighted orange. SNP, single nucleotide polymorphism; CRP, C-reactive protein; IL-6, interleukin 6; TNFα, tumour necrosis factor α; ICAM-1, intercellular adhesion molecule-1; LDL, low density lipoprotein; RH-PAT, reactive hyperaemia pulse amplitude tonometry; EPCs, endothelial progenitor cells; IMT, intima media thickness; ABI, ankle brachial index; LVH, left ventricular hypertrophy; ECG, electrocardiography; ACS, acute coronary syndrome; CAD, coronary artery disease; NCA, normal coronary arteries; TIA, transitory ischaemic attack; CT, computer tomography; MRI, magnet resonance imaging; GFR, glomerular filtration rate; BNP, brain natriuretic peptide. Adapted from Dzau et al. [15].

The study addressed the potential role of comprehensive systemic vascular assessment in patients presenting with symptoms suggestive of stable angina. Micro- and macrovascular structure and function relate to intermediate steps of cardiovascular disease progression [15]. In fact, with ageing, hypertension, cardiac and renal disease alterations in small and large arteries are now understood to be closely interrelated in a self-perpetuating cycle of remodelling and vascular damage [26]. We systematically evaluated these processes using validated clinical vascular markers as illustrated in Fig 4. Carotid plaque extent or microvascular endothelial function differentiated between patients with CAD and NCA in our study cohort. We have however not found any differences in reflection magnitude as well as the macrovascular parameters pulse wave velocity, C-IMT and carotid distensibility, between the two groups.

The latter macrovascular markers, despite their relation to intermediate steps of cardiovascular disease [15], lack discriminatory capacity in our cohort. This finding most likely relates to the similarity of traditional cardiovascular risk factors in CAD and NCA patients of our cohort. In particular blood pressure and age, two of the major determinants of C-IMT and arterial stiffness [26], were evenly distributed. We therefore assume that cardiovascular disease progression had only a small, non-detectable effect on these markers, explaining our finding.

Reports on microvascular endothelial function as measured by the RHI to identify CAD in cohorts of different origin showed ambiguous results [12–14]. RHI has been linked to different processes leading to angina symptoms, in particular cardiac microvascular dysfunction and cardiac macrovascular endothelial dysfunction [12], a precursor of atherosclerosis. Invasive assessment of the coronary microcirculation comes with the costs and risks associated with angiography. Migration of these techniques to the peripheral circulation with non-invasive methods enables repeated evaluation of measurements thought to closely mirror coronary artery microvascular function [17]. Patients with NCA and an abnormal stress test response often suffer from cardiac microvascular dysfunction [27], which is often associated with more widespread systemic microvascular disease [28]. In our study, however, we observed worse RHI values in the CAD patients with positive functional test compared to the NCA group. Although patients with NCA and a positive functional test can suffer from cardiac microcirculatory dysfunction [12], the difference in non-cardiac microvascular endothelial function was sufficient to differentiate between patients with CAD and NCA in our study. It therefore appears that the magnitude of endothelial dysfunction in CAD patients is greater than in NCA patients consistent with published literature [13]. In addition it should be borne in mind that NCA patients with angina symptoms represent a quite heterogeneous group [12].

Atherosclerosis is a systemic disease where carotid and coronary artery plaque coincide. Not only are plaque characteristics similar in both arterial beds [29], but carotid plaque and CAD extent [30] as well as CAD complexity [9] also correlate. Unsurprisingly, the presence of carotid plaque differentiated chest pain patients referred for coronary angiography with CAD from those with NCA in several cohort studies [7–11]. Consequently the European Society of Cardiology recommends carotid ultrasound in chest pain patients without known atherosclerotic disease [2]. However, similar to cohort studies investigating RHI [12–14], these studies did not assess the marker’s additive value to guideline conform assessment of pre-test probability [1,2] and were limited to a single aspect of the cardiovascular continuum.

Probability estimation is not only critical to implement a proper diagnostic work-up but also to improve the yield of diagnostic tests. For instance, our cohort had an average CAD likelihood of 64% according to the combined Diamond/Forrester and CASS data [1]. Exercise tolerance testing, with a sensitivity of 68% and a specificity of 77% [24], would produce a diagnostic yield of 84% (True Positive/ All Positives Test Results) in our cohort. In this setting a 10% increase of probability estimates through exclusion of NCA patients with <5% [1] or <15% [2] CAD likelihood from stress testing would provide a 5% increase in diagnostic yield.

Several methods for determination of pre-test probability have been described [1,2]. In our cohort only the algorithm by Pryor et al. [4,5] and the table by Genders et al. [2,25] showed statistical significant differences between CAD and NCA patients. According to the combined Diamond/Forrester and CASS data [1] our cohort had a CAD likelihood of 68% in the CAD group and 60% in the NCA group meaning that 32% or 60% of patients were incorrectly allocated. More sensitive means for identification of individuals with symptomatic CAD could therefore inform further diagnostic testing and prevent unnecessary invasive procedures.

In this study, the addition of RHI, carotid plaque score or their combination to an established pre-test probability algorithm [4,5] increased the c-statistic by 0.055, 0.105 and 0.136, respectively. Also, the combination of RHI and carotid plaque score in addition to pre-test probability values contributed significantly to logistic regression models predicting CAD. We therefore provided evidence that these measurements have the potential to improve CAD probability estimation. Also, assessment of carotid plaque, in particular absence of such with a negative predictive value of 80–81% in the literature [7,11] and 82% in our cohort, probably represents the best approach to pre-test probability improvement in clinical practice as a “rule-out test” due to its low cost, repeatability and wide availability.

The addition of RHI, carotid plaque score or their combination to the post-test probability value, based on pre-test probability [4,5] and stress test modality/result, increased the c-statistic by 0.120, 0.128 and 0.183, respectively. The approach produced results at least similar to pre-test probability estimations in our cohort and provides a feasible strategy to improve diagnostic yield in patients referred for coronary angiography.

Due to the retrospective study design patients differed from their first cardiology assessment in terms of medical management, percutaneous coronary intervention, exercise capacity and lifestyle modifications. As luminal obstruction of the left main stem ≥50% and/or at least one coronary artery stenosis ≥75% of the remaining coronary arteries as seen on coronary angiography was the inclusion criteria for patients with CAD, we also did not address the relationship between endothelial function or carotid plaque and non-flow limiting CAD. Previous studies have however shown similar RHI in patients with hemodynamically significant and non-significant coronary lesions [12] and in the iPOWER study investigating 339 middle aged to elderly women with angina symptoms and non-flow limiting CAD the use of the cut-off value RHI = 2.0 [31] lead to a similar sensitivity and specificity as in our study, 56% and 61% vs. 58% and 52%, respectively. Carotid plaque, however, occurs in obstructive and non-obstructive CAD, although more often in the former [11]. Also our study and others [8–14] only investigated patients referred for coronary angiography. A large percentage of patients presenting with chest pain, such as patients with low CAD likelihood or negative stress test results are therefore excluded from the analysis resulting in a degree of selection bias.

## Conclusions

Changes in both the systemic macro- and microcirculation are evident in patients with angina symptoms in whom CAD is confirmed at angiography. We identified carotid plaque and microvascular endothelial function as markers capable of differentiating patients who are found to have CAD from those with NCA. Both markers, alone or in combination, have the potential to improve pre- and post-test probability assessment for angiographyically significant CAD.

## Supporting information

S1 Fig Flowchart of the study design and recruitment process. CAD, coronary artery disease; NCA, normal coronary arteries; PWA, pulse wave analysis; SPECT, single positron emission computer tomography; EndoPAT, Endo-PAT2000 device (Itamar Medical Ltd., Caesarea, Israel).
(TIF)

S2 Fig Illustration of the used carotid plaque score. Areas with plaque burden are highlighted with arrows in B-mode common carotid artery images (A). The carotid plaque definition according to the Mannheim carotid IMT consensus is listed (B). The carotid plaque score equation is shown in C. Regarding the carotid pictures in panel A this leads to a carotid plaque score of 4.8 (number of affected vessel segments: 4, number of visualised vessel segments: 5).
(TIF)

S3 Fig Illustration of the distensibility coefficient. Depicted are relevant measurements on M-mode images of the common carotid artery (A) and the distensibility coefficient equation (B) as well as Cross-sectional B-mode image of the common carotid artery (C) in systole (left side) and diastole (right side). DC, distensibility coefficient; Dd, diastolic diameter; Ds, systolic diameter; SBP, systolic blood pressure; DBP, diastolic blood pressure.
(TIF)

S4 Fig Calculation of aortic forward and backward pressure waves. The central pulse wave and a corresponding triangular aortic flow wave [18] are shown in A. Equations for calculation of forward and backward pressure waves are given (A). B illustrates an aortic flow wave with corresponding forward and backward pressure waves. cPP, central pulse pressure; ESp, end-systolic pressure; P1, pressure at T1; Pf, forward pressure wave; Pb, backward pressure wave; Zc, aortic characteristic impedance; F, flow; T1, time at first inflection point; ED, time point marking the start of diastole (early diastole).
(TIF)

S1 Table Additional non-cardiac vascular markers. AIx, augmentation index; CAD, coronary artery disease; cDBP, central diastolic blood pressure; cPP, central pulse pressure; cSBP, central systolic blood pressure; NCA, normal coronary arteries; pPP, peripheral pulse pressure.
(PDF)

S1 File Supplementary methods. (DOC)

S1 Data Excel file containing the data contributing to the manuscript. (XLS)
