# Supplementary figures and images for: Evaluation of the systemic micro- and macrovasculature in stable angina: A case-control study
Source: PLoS One. 2017 May 25;12(5):e0178412. doi: 10.1371/journal.pone.0178412 (PMC5444845; doi:10.1371/journal.pone.0178412)

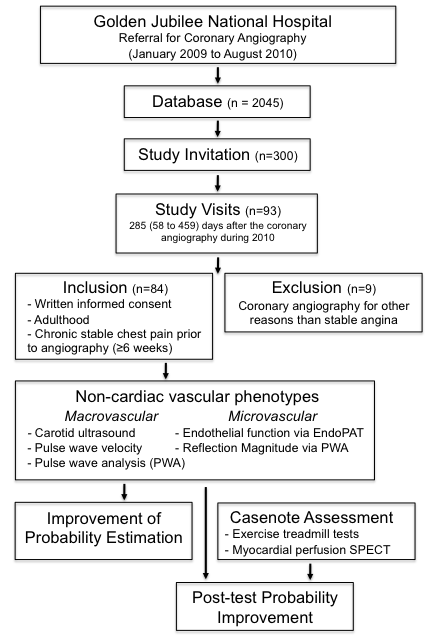

Supplement: S1 Fig — CAD, coronary artery disease; NCA, normal coronary arteries; PWA, pulse wave analysis; SPECT, single positron emission computer tomography; EndoPAT, Endo-PAT2000 device (Itamar Medical Ltd., Caesarea, Israel). (TIF) [file pone.0178412.s001.tif]

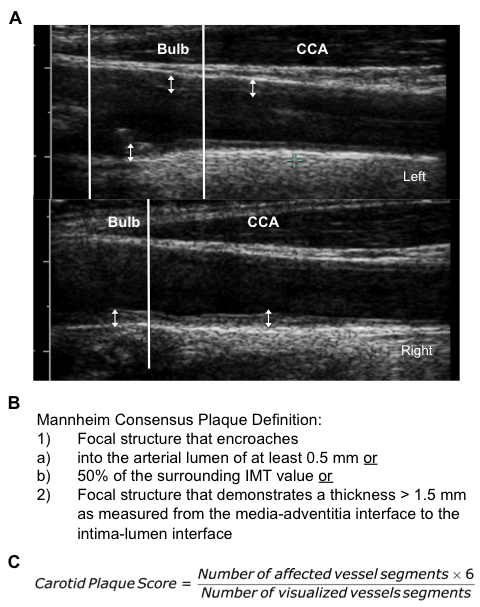

Supplement: S2 Fig — Areas with plaque burden are highlighted with arrows in B-mode common carotid artery images (A). The carotid plaque definition according to the Mannheim carotid IMT consensus is listed (B). The carotid plaque score equation is shown in C. Regarding the carotid pictures in panel A this leads to a carotid plaque score of 4.8 (number of affected vessel segments: 4, number of visualised vessel segments: 5). (TIF) [file pone.0178412.s002.tif]

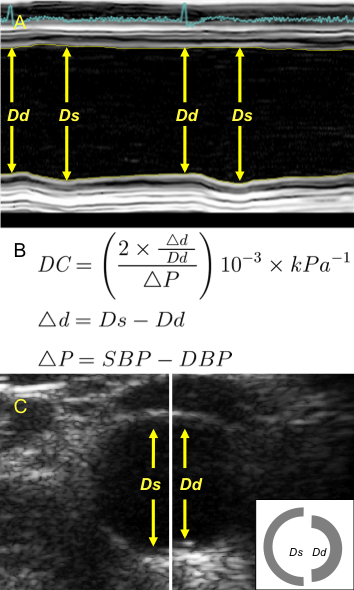

Supplement: S3 Fig — Depicted are relevant measurements on M-mode images of the common carotid artery (A) and the distensibility coefficient equation (B) as well as Cross-sectional B-mode image of the common carotid artery (C) in systole (left side) and diastole (right side). DC, distensibility coefficient; Dd, diastolic diameter; Ds, systolic diameter; SBP, systolic blood pressure; DBP, diastolic blood pressure. (TIF) [file pone.0178412.s003.tif]

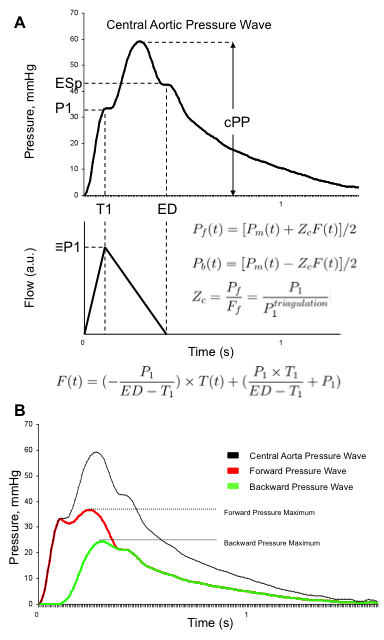

Supplement: S4 Fig — The central pulse wave and a corresponding triangular aortic flow wave [18] are shown in A. Equations for calculation of forward and backward pressure waves are given (A). B illustrates an aortic flow wave with corresponding forward and backward pressure waves. cPP, central pulse pressure; ESp, end-systolic pressure; P1, pressure at T1; Pf, forward pressure wave; Pb, backward pressure wave; Zc, aortic characteristic impedance; F, flow; T1, time at first inflection point; ED, time point marking the start of diastole (early diastole). (TIF) [file pone.0178412.s004.tif]
