# Supplementary material for: Evaluation of the systemic micro- and macrovasculature in stable angina: A case-control study
Source: PLoS One. 2017 May 25;12(5):e0178412. doi: 10.1371/journal.pone.0178412 (PMC5444845; doi:10.1371/journal.pone.0178412)
Supplement: S1 Table — AIx, augmentation index; CAD, coronary artery disease; cDBP, central diastolic blood pressure; cPP, central pulse pressure; cSBP, central systolic blood pressure; NCA, normal coronary arteries; pPP, peripheral pulse pressure. (PDF) [file pone.0178412.s005.pdf]

	CAD (n=43)	NCA (n=41)	*P*-Value
pPP, mmHg	54 [48; 63]	56 [47; 64]	0.813
AIx, %	26±9	28±10	0.308
Supine cSBP, mmHg	124±16	127±13	0.386
Supine cDBP, mmHg	76±10	79±9	0.083
Supine cPP, mmHg	47 [41; 52]	44 [38; 56]	0.507
Cross-sectional compliance, $m^2 \cdot kPa^{-1} \cdot 10^{-7}$	9.5 [7.0; 12.7]	8.7 [6.5; 11.6]	0.386
