# Supplementary material for: Evaluation of the systemic micro- and macrovasculature in stable angina: A case-control study
Source: PLoS One. 2017 May 25;12(5):e0178412. doi: 10.1371/journal.pone.0178412 (PMC5444845; doi:10.1371/journal.pone.0178412)
Supplement: S1 File — (DOC) [file pone.0178412.s006.doc]

**Evaluation of the systemic micro- and macrovasculature in stable angina: a case-control study**

Ulf Neisius^1^, Erin Olson^1^, Sabrina H Rossi^1^, Hagar A Ibrahim^1^, Gemma Currie^1^, Anna F Dominiczak^1^, Christian Delles^1^*

^1^ BHF Glasgow Cardiovascular Research Centre, Institute of Cardiovascular and Medical Sciences, University of Glasgow, 126 University Place, Glasgow G12 8TA, UK

**S1 File. Supplementary Methods**

**Carotid ultrasound**

**Carotid intima-media thickness**

An increased carotid intima-media thickness (C-IMT) mostly represents a marker of early atherosclerosis. Measurement of carotid intima-media C-IMT was performed by ultrasonography (Acuson Sequoia C512, Siemens, Erlangen, Germany) with an 8 MHz linear-array transducer. The anterior (near) and posterior (far) walls of the left and right common carotid arteries were displayed as two bright white lines separated by a hypoechogenic space in anterolateral, posterlateral and mediolateral directions on longitudinal, two-dimensional ultrasound images. The distance between the leading edge of the first bright line of the far wall (lumen-intima interface) and the leading edge of the second bright line (media-adventitia interface) indicates the C-IMT. For the near wall, the distance between the trailing edge of the first bright line and the trailing edge of the second bright line at the near wall provides the best estimate of the near wall C-IMT. The carotid media thickness was measured in the far wall, 1 cm proximal of the carotid bulb in a plaque free region in accordance with the Mannheim consensus [25]. ECG signals were stored simultaneously to define the systolic and diastolic phase of the cardiac cycle. Offline measurements were performed semi-automatically at end diastole on B-mode images using Image-Pro Plus software, version 3.0 (Media Cybernetics, Bethesda, USA). Depending on image quality up to six different measurements corresponding to the recording directions on both common carotid arteries were used to calculate the average C-IMT.

**Carotid plaque**

Carotid plaque represents a marker of progressed arterial atherosclerosis succeeding increased C-IMT values. To assess carotid plaque semi-quantitatively, longitudinal offline B-mode common carotid artery images in anterolateral, mediolateral and posterolateral direction were assessed for plaque presence in the near and far wall of the carotid arteries. Corresponding to the Mannheim carotid IMT consensus [19] plaque was defined as a “focal structure that encroaches into the arterial lumen of at least 0.5 mm or 50% of the surrounding C-IMT value or demonstrates a thickness > 1.5 mm as measured from the media-adventitia interface to the intima-lumen interface”. As published by Hollander et al. [20] and van der Meer et al. [21] the plaque score had values from 0 to 6. The number of sites with a detected plaque (left- and right-sided common carotid artery, bifurcation and internal carotid artery) is divided by the total number of sites with an available ultrasonographic image and then multiplied by six (S2 Fig). The size or the extent of the lesions was not evaluated. Plaque quantification was only conducted if ≥2 out of 6 sites were imaged appropriately. The corresponding carotid plaque score is illustrated in S2 Fig.

**Carotid distensibility**

Carotid distensibility is a marker of vascular stiffness. Common carotid distensibility was assessed with the study participants in supine position, with the head tilted slightly to the contralateral side. Diameter changes in the common carotid arteries were recorded during the cardiac cycle to assess carotid distensibility. Perpendicular to the common carotid artery wall, as visualised on ultrasonographic B-mode, an M-mode line was recorded over several heart cycles. The first bright line of the far wall and the trailing edge of the second bright line of the near wall were defined as the vessel lumen borders. To avoid a distensibility reduction, no pressure was applied exceeding the weight of the 8 MHz linear-array transducer. Minimal and maximal carotid diameters were measured offline on the right and left common carotid artery at a plaque-free site, 1 cm below the bulb with Image-Pro Plus software, version 3.0 (Media Cybernetics, Bethesda, USA). The minimum and maximum diameters during a heart cycle were defined as diastolic and systolic diameters, respectively. Depending on picture quality, M-mode pictures from anterolateral, mediolateral and posterolateral directions were evaluated. The measurements of the left and right common carotid artery were averaged and subsequently the average of both sides was used for calculation of the distensibility coefficient. The distensibility coefficient was calculated according to the following equation [S1]:

Distensibility coefficient [10^-3^/kPa] = (2ΔD/D) / ΔP

In this equation ΔD represents the diameter change of common carotid artery during systole, D represents the end-diastolic diameter and ΔP stands for the pulse pressure. The distensibility coefficient is illustrated in S3 Fig. Due to anatomical proximity the central blood pressure measurements as provided by PWA were used in the calculation. As a further index of artery stiffness the cross-sectional compliance [S1] was calculated by the following equation:

Cross-sectional Compliance [m^2^ * kPa^-1^ * 10^-7^] = π * (D_systolic_ ^2^-D^2^)/(4ΔP)

In this equation D_systolic_ represents the maximum systolic diameter, π is approximately 3.14159 and further abbreviations are identical to the distensibility coefficient equation.

**Pulse wave analysis**

For pulse wave analysis participants were rested for 20 minutes in supine position at a temperature 23-24 °C. Radial arterial pressure waveforms were recorded with a Millar piezo-resistive pressure transducer (Millar SPT 301, Millar Instruments, Houston, US) coupled to a SphygmoCor device (AtCor Medical, Sydney, Australia). The corresponding central (ascending aortic) waveform was generated by the SphygmoCor software (version 7.0) using a generalised and validated [S2] transfer function. Supine peripheral blood pressure measurements were used for calibration. The central augmentation index (AIx) was calculated from the aortic pressure waveforms, where AIx is equal to the ratio of pressure augmentation and pulse pressure. Aortic systolic and diastolic blood pressure were derived from the aortic pressure waveform under the assumption of a constant mean arterial pressure. The mean arterial pressure was calculated with the integration of the radial waveform. Only measurements of good quality, defined as an operator index ≥80 determined by the software of the SphygmoCor device were used. Central pulse pressure was defined as the difference between aortic systolic and diastolic blood pressure. Peripheral pulse pressure was defined as the difference between brachial systolic and diastolic blood pressure in supine position.

**Reflection magnitude**

Reflection magnitude represents predominantly the extent of pressure wave reflection by small resistance vessels. Under the assumption of a triangular aortic flow wave, the reflection magnitude can be calculated based on pulse wave analysis parameters. To quantify the maximum of the forward and backward pressure waves in the aortic root the triangulation method as reported by Westerhof et al. [18] was used. The parameters shown in S4 Fig were used to calculate the forward and backward pressure waves based on central pressure curve measured by the Sphygmocor device. The formula [S3] for the forward and backward pressure amplitudes as well as the related waves are depicted in S4 Fig. The shape of the triangle can be reconstructed with pressure/time ratios extracted from the central pulse wave. To adjust for the magnitude of the pressure wave the reflection magnitude was calculated as the ratio of the backward and forward pressure amplitudes.

**Pulse wave velocity**

Carotid-femoral pulse wave velocity (PWV) is a maker of aortic stiffness (descending thoracic aorta, abdominal aorta, and iliac arteries). PWV was measured using the SphygmoCor device according to the manufacturers’ protocol. Participants were studied in the supine position after resting more than 5 minutes. Supine brachial systolic and diastolic blood pressures were obtained with the use of an oscillometric device. Three surface electrodes were positioned on both arms and the abdomen for continuous ECG tracing. The distances “common carotid artery to suprasternal notch” (d_ster-car_) and “femoral artery to suprasternal notch” (d_ster-fem_) were measured with a measuring tape. To avoid measurement errors the linear distance instead of the body surface distance was used. The travel distance was estimated by the subtracted distance method [S4], as shown in the following equation:

Travel Distance _subtracted distance_ = d_ster-fem_ - d_ster-car_.

Femoral and carotid pulse wave tracings were obtained consecutively with the Millar pressure transducer in parallel to ECG recordings for 10 seconds. Recordings were performed consecutively on the right common carotid artery and the right femoral artery in close proximity to the inguinal ligament. Only in cases where right-sided measurements were insufficient, the left common carotid artery or the femoral artery was assessed. Data was analyzed by a computerized algorithm, which operator independent and automatically calculated the carotid-femoral PWV. In particular, PWV was determined from time delay differences between the QRS complex onset on an ECG and the upstroke of the pressure tracing measured at the femoral and carotid arteries. PWV was determined from the foot-to-foot time interval of waveforms. Measurements were rejected if the standard deviation of single heart beat intervals was bigger than 10% during the recording period.

**CAD probability estimation**

To calculate the probability of significant CAD according to Pryor et al. [4,5] the following criteria were used. Pathologic Q-waves were defined according to current guidelines [S5]. Hyperlipidaemia was defined as either fasting cholesterol of ≥6.5 mmol/l or a history of hyperlipidemia. For probability calculation smoking status was considered positive if currently or during the last 12 months a minimum of 10 cigarettes per day were consumed. The diagnoses of diabetes mellitus, hypertension, cerebral vascular events and positive family history were based on self-reported information acquired during the study visits or medical records.

**Supplementary References**

S1. [Reneman RS](http://www.ncbi.nlm.nih.gov/pubmed/?term=Reneman RS%5BAuthor%5D&cauthor=true&cauthor_uid=3523921), [van Merode T](http://www.ncbi.nlm.nih.gov/pubmed/?term=van Merode T%5BAuthor%5D&cauthor=true&cauthor_uid=3523921), [Hick P](http://www.ncbi.nlm.nih.gov/pubmed/?term=Hick P%5BAuthor%5D&cauthor=true&cauthor_uid=3523921), [Muytjens AM](http://www.ncbi.nlm.nih.gov/pubmed/?term=Muytjens AM%5BAuthor%5D&cauthor=true&cauthor_uid=3523921), [Hoeks AP](http://www.ncbi.nlm.nih.gov/pubmed/?term=Hoeks AP%5BAuthor%5D&cauthor=true&cauthor_uid=3523921). Age-related changes in carotid artery wall properties in men. Ultrasound Med Biol. 1986;12:465-71.

S2. Pauca AL, O'Rourke MF, Kon ND. Prospective evaluation of a method for estimating ascending aortic pressure from the radial artery pressure waveform. Hypertension. 2001;38:932-7.

S3. [Murgo JP](http://www.ncbi.nlm.nih.gov/pubmed/?term=Murgo JP%5BAuthor%5D&cauthor=true&cauthor_uid=7438386), [Westerhof N](http://www.ncbi.nlm.nih.gov/pubmed/?term=Westerhof N%5BAuthor%5D&cauthor=true&cauthor_uid=7438386), [Giolma JP](http://www.ncbi.nlm.nih.gov/pubmed/?term=Giolma JP%5BAuthor%5D&cauthor=true&cauthor_uid=7438386), [Altobelli SA](http://www.ncbi.nlm.nih.gov/pubmed/?term=Altobelli SA%5BAuthor%5D&cauthor=true&cauthor_uid=7438386). Manipulation of ascending aortic pressure and flow wave reflections with the Valsalva maneuver: relationship to input impedance. Circulation. 1981;63:122-32.

S4. [Vermeersch SJ](http://www.ncbi.nlm.nih.gov/pubmed/?term=Vermeersch SJ%5BAuthor%5D&cauthor=true&cauthor_uid=19898251), [Rietzschel ER](http://www.ncbi.nlm.nih.gov/pubmed/?term=Rietzschel ER%5BAuthor%5D&cauthor=true&cauthor_uid=19898251), [De Buyzere ML](http://www.ncbi.nlm.nih.gov/pubmed/?term=De Buyzere ML%5BAuthor%5D&cauthor=true&cauthor_uid=19898251), [Van Bortel LM](http://www.ncbi.nlm.nih.gov/pubmed/?term=Van Bortel LM%5BAuthor%5D&cauthor=true&cauthor_uid=19898251), [Gillebert TC](http://www.ncbi.nlm.nih.gov/pubmed/?term=Gillebert TC%5BAuthor%5D&cauthor=true&cauthor_uid=19898251), [Verdonck PR](http://www.ncbi.nlm.nih.gov/pubmed/?term=Verdonck PR%5BAuthor%5D&cauthor=true&cauthor_uid=19898251), [Laurent S](http://www.ncbi.nlm.nih.gov/pubmed/?term=Laurent S%5BAuthor%5D&cauthor=true&cauthor_uid=19898251), [Segers P](http://www.ncbi.nlm.nih.gov/pubmed/?term=Segers P%5BAuthor%5D&cauthor=true&cauthor_uid=19898251), [Boutouyrie P](http://www.ncbi.nlm.nih.gov/pubmed/?term=Boutouyrie P%5BAuthor%5D&cauthor=true&cauthor_uid=19898251). Distance measurements for the assessment of carotid to femoral pulse wave velocity. J Hypertens. 2009;27:2377-85.

S5. Thygesen K, Alpert JS, White HD, Jaffe AS, Apple FS, Galvani M, Katus HA, Newby LK, Raykilde J, Chaitman B, Clemmensen PM, Dellborg M, Hod H, Porela P, Underwood R, Bax JJ, Beller GA, Bonow R, Van der Wall EE, Bassand JP, Wijns W, Ferguson TB, Steg PG, Uretsky BF, Williams DO, Armstrong PW, Antman EM, Fox KA, Hamm CW, Ohman EM, Simoons ML, Poole-Wilson PA, Gurfinkel EP, Lopez-Sendon JL, Pais P, Mendis S, Zhu JR, Wallentin LC, Fernández-Avilés F, Fox KM, Parkhomenko AN, Priori SG, Tendera M, Voipio-Pulkki LM, Vahanian A, Camm AJ, De Caterina R, Dean V, Dickstein K, Filippatos G, Funck-Brenatano C, Hellemans I, Kristensen SD, McGregor K, Sechtem U, Silber S, Tendera M, Widimsky P, Zamorano JL, Morais J, Brener S, Harrington R, Morrow D, Lim M, Martinez-Rios MA, Steinhubl S, Levine GN, Gibler WB, Goff D, Tubaro M, Dudek D, Al-Attar N. Universal definition of myocardial infarction. Circulation. 2007;116:2634-53.

Supplementary Figures (S1-S5) are provided in separate files
